# Identification of Senescence-Associated Biomarkers in Diabetic Glomerulopathy Using Integrated Bioinformatics Analysis

**DOI:** 10.1155/2024/5560922

**Published:** 2024-01-23

**Authors:** Li Zhang, Zhaoxiang Wang, Fengyan Tang, Menghuan Wu, Ying Pan, Song Bai, Bing Lu, Shao Zhong, Ying Xie

**Affiliations:** ^1^Department of Endocrinology, The Second Affiliated Hospital of Soochow University, Suzhou 215008, Jiangsu, China; ^2^Department of Endocrinology, The First People's Hospital of Kunshan, Kunshan 215300, Jiangsu, China; ^3^Department of Cardiology, Xuyi People's Hospital, Xuyi 211700, Jiangsu, China

## Abstract

**Background:**

Cellular senescence is thought to play a significant role in the onset and development of diabetic nephropathy. The goal of this study was to explore potential biomarkers associated with diabetic glomerulopathy from the perspective of senescence.

**Methods:**

Datasets about human glomerular biopsy samples related to diabetic nephropathy were systematically obtained from the Gene Expression Omnibus database. Hub senescence-associated genes were investigated by differential gene analysis and Least Absolute Shrinkage and Selection Operator analysis. Cluster analysis was employed to identify senescence molecular subtypes. A single-cell dataset was used to validate the above findings and further evaluate the senescence environment. The relationship between these genes and the glomerular filtration rate was explored based on the Nephroseq database. These gene expressions have also been explored in various kidney diseases.

**Results:**

Twelve representative senescence-associated genes (VEGFA, IQGAP2, JUN, PLAT, ETS2, ANG, MMP14, VEGFC, SERPINE2, CXCR2, PTGES, and EGF) were finally identified. Biological changes in immune inflammatory response, cell cycle regulation, metabolic regulation, and immune microenvironment have been observed across different molecular subtypes. The above results were also validated based on single-cell analysis. Additionally, we also identified several significantly altered cell communication pathways, including COLLAGEN, PTN, LAMININ, SPP1, and VEGF. Finally, almost all these genes could well predict the occurrence of diabetic glomerulopathy based on receiver operating characteristic analysis and are associated with the glomerular filtration rate. These genes are differently expressed in various kidney diseases.

**Conclusion:**

The present study identified potential senescence-associated biomarkers and further explored the heterogeneity of diabetic glomerulopathy that might provide new insights into the diagnosis, assessment, management, and personalized treatment of DN.

## 1. Introduction

In recent decades, the prevalence of both type 1 diabetes mellitus (T1DM) and type 2 diabetes mellitus (T2DM) has rapidly increased worldwide, leading to a corresponding rise in microvascular and macrovascular diabetes-related complications [1]. Studies estimate that around 30-40% of patients with diabetes mellitus will develop diabetic nephropathy (DN), and almost half of these individuals will ultimately progress to end-stage renal disease (ESRD) [2, 3]. Current therapeutic approaches for managing DN involve controlling blood pressure and glucose levels and administering angiotensin receptor blockers (ARBs) and angiotensin-converting enzyme inhibitors [2]. However, despite these interventions, they have shown limited effectiveness in preventing the progression of DN [2, 4].

Senescent cells are characterized by a state of permanent growth arrest and altered secretory phenotype [5]. The accumulation of these cells is commonly observed in the kidney and other organs during the aging process and in response to injury [6]. The mechanisms underlying the relationship between senescence and DN are multifaceted and intricate [7]. It has been postulated that senescence leads to a decline in renal function, resulting in a reduced ability to eliminate waste from the bloodstream and an increased susceptibility to damage from free radicals and circulating toxins [8]. Immune system alterations associated with senescence, such as impaired immunity and chronic low-grade inflammation, may contribute to the development of DN [8, 9]. Dysregulated metabolism, especially mitochondrial dysfunction, and oxidative stress, may play a crucial role in the pathogenesis of both aging and DN, promoting cellular damage [10, 11]. The identification and characterization of senescence-associated biomarkers in the context of diabetic glomerulopathy have the potential to aid in providing new insights into the pathogenesis of this disease and the development of novel therapeutic interventions [4, 12].

In this study, we identified several hub biomarkers related to diabetic glomerulopathy from the perspective of senescence. The senescence-associated molecular subtypes in diabetic glomerulopathy were further explored by clustering analysis. Single-cell transcriptomics was used to evaluate this finding and further investigate cellular heterogeneity and biological changes in diabetic glomerulus cells in different senescence environments.

## 2. Methods

### 2.1. Differential Gene Analysis and Senescence-Associated Genes

**Figure 1:**
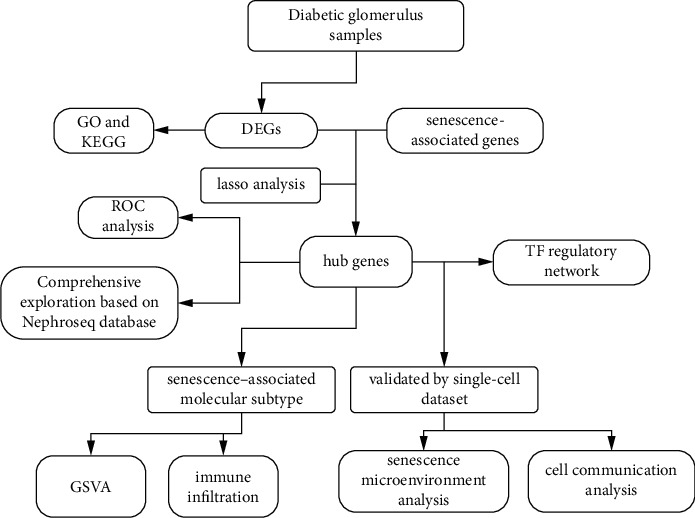
Flow diagram of this study.

The framework we selected in this study is shown in Figure 1. Two DN datasets (GSE96804 and GSE30528) were collected and integrated from the Gene Expression Omnibus (GEO) database, encompassing 50 glomerular tissue samples from DN patients and 33 glomerular tissue samples from control human kidneys [13]. The specific details of these sequencing glomerular tissue samples, including inclusion and exclusion criteria, are well articulated in the original article by the dataset uploader (https://www.ncbi.nlm.nih.gov/geo/) [14, 15]. Batch effects were corrected using the ComBat function in the sva R package [16]. Differential gene analysis was conducted using the Limma R package, with selection criteria for differentially expressed genes (DEGs) being a *P* value < 0.05 and |log2 (fold change)| > 0.5 [17]. Annotation of the DEGs was performed using Gene Ontology (GO) enrichment and Kyoto Encyclopedia of Genes and Genomes (KEGG) pathway analysis [18]. GO consists of biological processes (BP), molecular functions (MF), and cellular components (CC). An intersection analysis was conducted between the DEGs and senescence-associated genes, using the set of 125 genes previously defined by Saul et al. [5]. The Spearman correlation coefficients of these intersection genes were calculated, and KEGG pathway analysis was also subsequently performed.

### 2.2. Identification of Hub Genes and Molecular Subtypes

Hub senescence-associated genes were identified by performing Least Absolute Shrinkage and Selection Operator (LASSO) analysis based on the intersection genes. LASSO analysis is a commonly used method for variable screening, as it constructs a penalty function that generates a more refined model, compressing the coefficient size and enabling more accurate identification of relevant genes [19]. The JASPAR database was employed to predict the potential transcription factors (TFs) that regulate hub genes, and subsequently construct a TF-gene network. JASPAR is a publicly accessible and cost-free database for TFs that provides comprehensive information on the binding sites and mechanisms of TFs to DNA [20]. Based on the expression profiles of hub genes in the diabetic glomerulopathy samples, we used the ConsensusClusterPlus R package with partitioning around medoid (PAM) clustering and a sampling fraction of 0.8 to classify distinct senescence-associated molecular subtypes in diabetic glomerulopathy. ConsensusClusterPlus is an R package that provides a consensus clustering method for analyzing various types of biological data, such as gene expression and proteomic data [21]. To assess the expression distribution of KEGG signaling pathways, gene set variation analysis (GSVA) was conducted [22]. Furthermore, the proportions of immune cells in diabetic glomerulopathy samples were determined via CIBERSORT, and any differences in immune cell abundance between senescence-associated molecular subtypes were compared using a significance threshold of *P* value < 0.05. CIBERSORT is a computational tool that analyzes the composition of immune cells by estimating the relative abundance of different immune cell types from gene expression data [23].

### 2.3. Single-Cell Data Analysis

The scRNA-seq dataset GSE218563 obtained from diabetic kidney cells was processed and analyzed using the Seurat R package [24]. The dataset GSE218563 contains kidney samples from a total of 16 mice, including both male and female BTBR ob/ob (DN) and BTBR WT (control) mice at ages 06 and 12 weeks. The onset stage of DN was defined as 06 weeks of age, while the early stage of DN was defined as 12 weeks of age. More detailed information about scRNA-seq dataset GSE218563 is seen in https://www.ncbi.nlm.nih.gov/geo/ [25]. Quality control of cells was implemented based on the previous criteria [25]. To mitigate batch effects, the Harmony algorithm was applied, which projects cells into a shared embedding where they are grouped by cell type rather than dataset-specific conditions [26]. Clustering results were visualized using Uniform Manifold Approximation and Projection (UMAP), and cell clusters were annotated according to relevant literature [25, 27].

A specific senescence gene set to diabetic kidney cells was constructed based on the identified hub genes. The extent of cellular senescence was assessed through the AddModuleScore function in the Seurat R package. The Seurat AddModuleScore function is used to calculate and add module scores to each cell in a Seurat object, which can be used to assess the activity of gene modules or pathways within individual cells. The degree of cellular senescence was compared between diabetic and normal glomerular cells at 06 and 12 weeks, respectively. The Seurat FindMarker function was then employed to identify DEGs between glomerular cell clusters with high and low senescence scores. Subsequently, KEGG analysis was performed to investigate the biological pathways associated with these DEGs.

The CellChat platform was employed to infer cell-cell communication between different types of kidney glomerular cells. CellChat is a computational framework that enables the analysis of cell-cell communication networks using single-cell RNA sequencing data. It allows researchers to explore the interactions between different cell types and understand the communication dynamics within a complex biological system [28]. The Mouse database managed by CellChat, which comprises “secretory signaling,” “cell-cell contact,” and “ECM-receptor,” was utilized. The intensity, pathways, and ligand-receptor interactions of communication between high and low glomerular senescence scores were compared.

### 2.4. Clinical Value Evaluation and External Database Analysis

To evaluate the accuracy of predicting diabetic glomerulopathy based on the expression of hub senescence-associated genes, a receiver operating characteristic (ROC) analysis was performed using the R package pROC [29]. Additionally, based on the Nephroseq v5 online platform (https://nephroseq.com/), Spearman correlation analysis was conducted to investigate the relationship between gene expression levels and the glomerular filtration rate (GFR) in DN. The expression levels of these hub genes in the glomeruli were also measured under different types of kidney diseases (arterial hypertension, focal segmental glomerulosclerosis, lgA nephropathy, lupus nephritis, membranous glomerulonephropathy, minimal change disease, thin basement membrane disease, vasculitis, and DN). The sample information for different types of kidney diseases, including sample size, source, and inclusion and exclusion criteria, can all be accessed in the Nephroseq database. The Nephroseq database is a specialized database designed for research related to kidney diseases, which incorporates gene expression datasets from multiple sources, including invasive and noninvasive human urine, plasma, and kidney biopsy samples [30].

## 3. Results

### 3.1. DEGs and Senescence-Associated Genes

**Figure 2:**
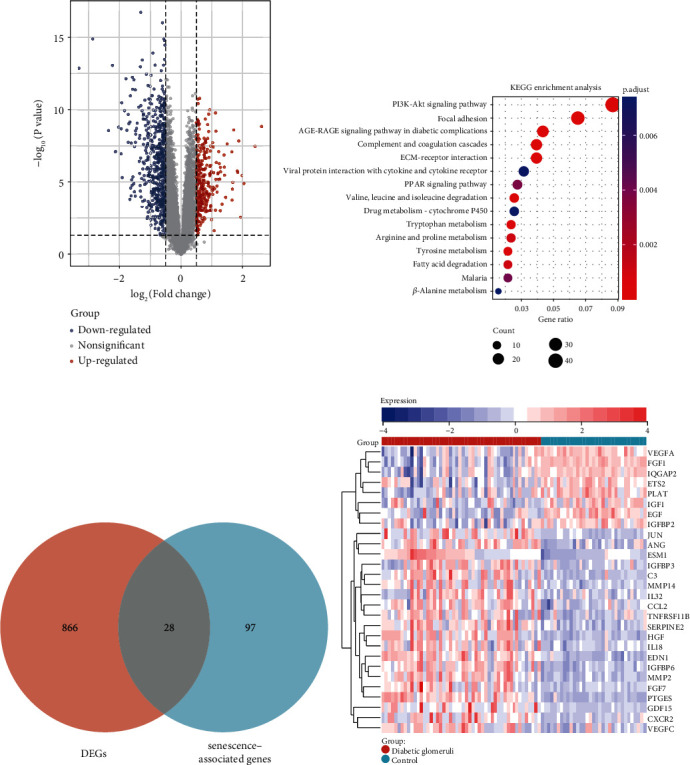
Senescence-associated genes in diabetic glomerulopathy. (a) Volcanic map of DEGs. (b) Bar plot of KEGG enrichment results for DEGs. (c) Venn diagrams of overlapping genes. (d) Heat map based on the expression of overlapping genes in diabetic glomerulopathy.

A differential gene analysis was performed on an integrated dataset (GSE96804 + GSE30528), resulting in the identification of 894 DEGs based on screening criteria (Figure 2(a)). The KEGG analysis of these DEGs showed that the PI3K-Akt signaling pathway, focal adhesion, and AGE-RAGE signaling pathway in a diabetic complication were relatively significant pathways (Figure 2(b)). GO analysis also revealed relevant biological changes involved in diabetic glomerulopathy (Attachment 1). By intersecting the DEGs with senescence-associated genes, a total of 28 common genes were obtained (Figure 2(c)). Among these genes, 20 genes (JUN, ANG, ESM1, IGFBP3, C3, MMP14, IL32, CCL2, TNFRSF11B, SERPIE2, HGF, IL18, EDN1, IGFBP6, MMP2, FGF7, PTGES, GDF15, CXCR2, and VEGFC) were upregulated and 8 genes (VEGFA, FGF1, IQGAP2, ETS2, PLAT, IGF1, EGF, and IGFBP2) were downregulated in the diabetic glomerulopathy group compared to the normal control group (Figure 2(d)).

### 3.2. LASSO Analysis and Identification of Hub Genes

**Figure 3:**
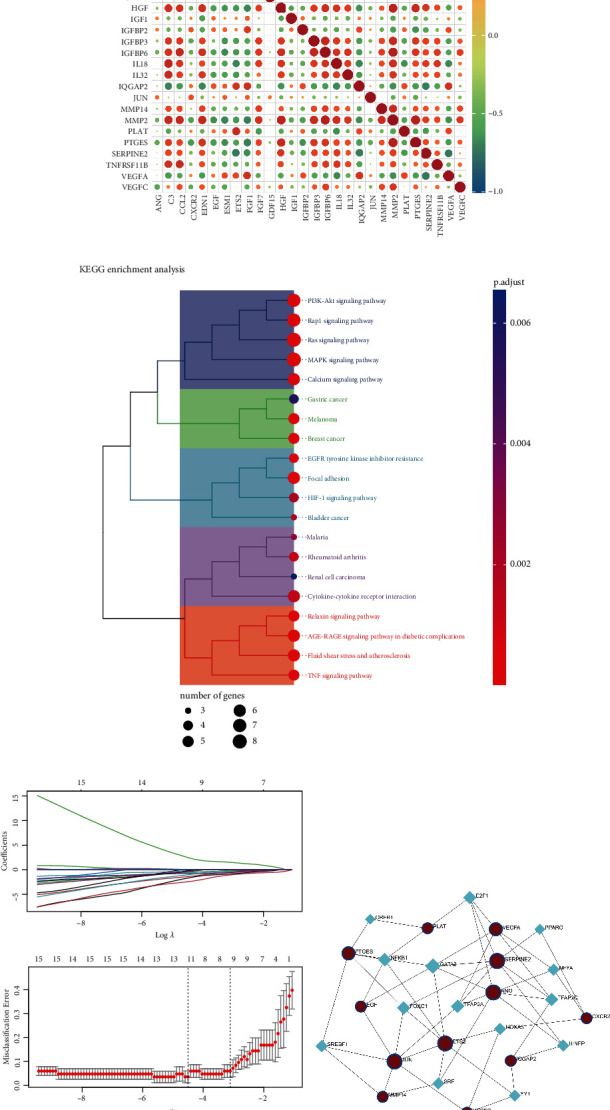
Identification of hub senescence-associated genes. (a) Correlation analysis between expressions of overlapping genes. (b) Bar plot of KEGG enrichment results for overlapping genes. (c) LASSO regression coefficient graph and cross-validation graph based on penalty term. (d) Regulatory network diagram of TFs according to hub genes.

The expression levels of these genes are closely correlated with each other (Figure 3(a)). The KEGG analysis found that they primarily involve biological alterations related to cellular growth, proliferation, apoptosis, differentiation, metabolism, and energy homeostasis (Figure 3(b)). To further select variables from the 28 genes, LASSO regression analysis was performed. The corresponding coefficients of these 28 genes in the LASSO regression model are shown in Figure 3(c) for various penalty parameters. The former *λ* (*λ*. 1se, 12 genes) was selected due to its more accurate model than the later *λ* (*λ*. min, 9 genes). Ultimately, 12 hub senescence-related genes (VEGFA, IQGAP2, JUN, PLAT, ETS2, ANG, MMP14, VEGFC, SERPINE2, CXCR2, PTGES, and EGF) were identified. In additional, TFs that regulate hub genes predicted based on the JASPAR database are shown in Figure 3(d).

### 3.3. Senescence-Associated Molecular Subtypes and Immune Infiltration

**Figure 4:**
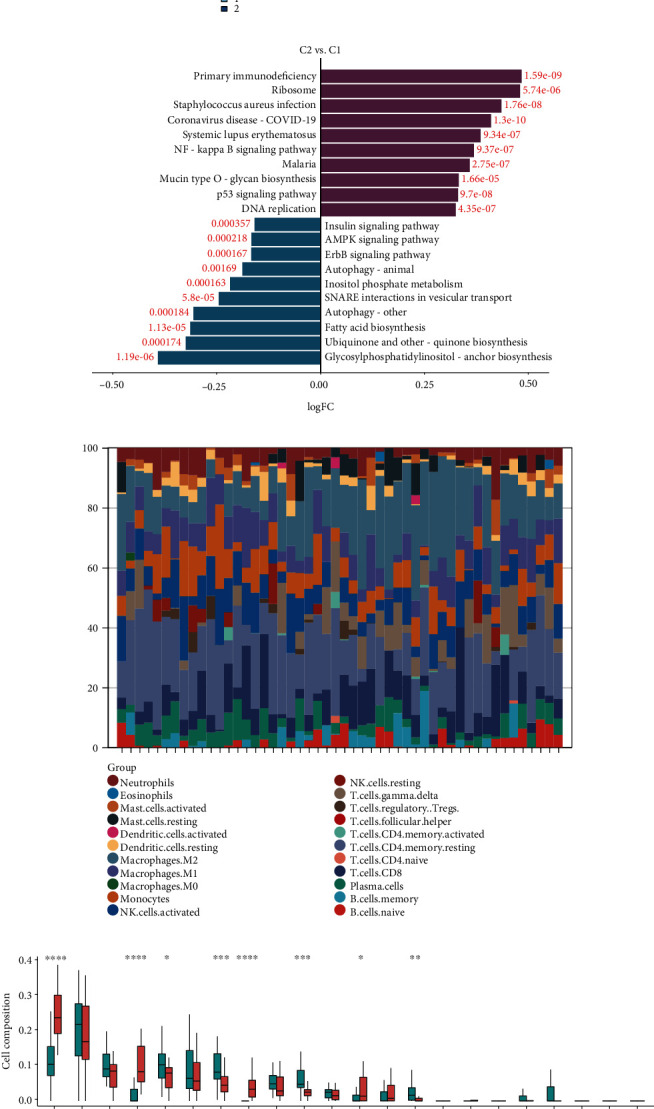
Senescence-associated molecular subtype analysis. (a) Consensus clustering to divide subtypes. (b) Bar chart to show the most significant pathways (upregulation and downregulation). (c) Bar proportions for each diabetic glomerulopathy sample. (d) Proportions of 22 types of immune cells in C1 and C2 subtypes (^∗^*P* value < 0.05, ^∗∗^*P* value < 0.01, and ^∗∗∗^*P* value < 0.001).

To further elucidate the potential biological mechanisms of these genes in diabetic glomerulopathy, we conducted clustering analysis of twelve hub senescence-associated genes to classify diabetic glomerulopathy samples into two subtypes (C1 and C2), as depicted in Figure 4(a). The top 10 upregulated pathways and the top 10 downregulated pathways are displayed in Figure 4(b). The most prominent biological changes observed in subtype C2 mainly involve cellular immune response, inflammatory response, and cell cycle regulation. In contrast, subtype C1 is primarily associated with metabolic processes that also involve cell proliferation, differentiation, and cell death, such as the ErbB signaling pathway.

By analyzing the proportion of immune cells in each sample, we compared the changes in the relative proportions of 22 immune cells between the C1 and C2 subgroups (Figure 4(c)). Our results showed that four types of immune cells (M2 macrophages, gamma-delta T cells, resting Mast cells, and memory B cells) infiltrated more in the C2 subtype, while activated NK cells, monocytes, neutrophil T cells, and activated Mast cells infiltrated less in the C2 subtype when compared with the C1 subtype. However, there were no statistically significant differences in the abundance of other types of immune cells between the C1 and C2 subtypes (Figure 4(d)). These above findings might suggest that the C2 subtype may represent an advanced senescence stage of diabetic glomerulopathy.

### 3.4. Single-Cell Analysis

**Figure 5:**
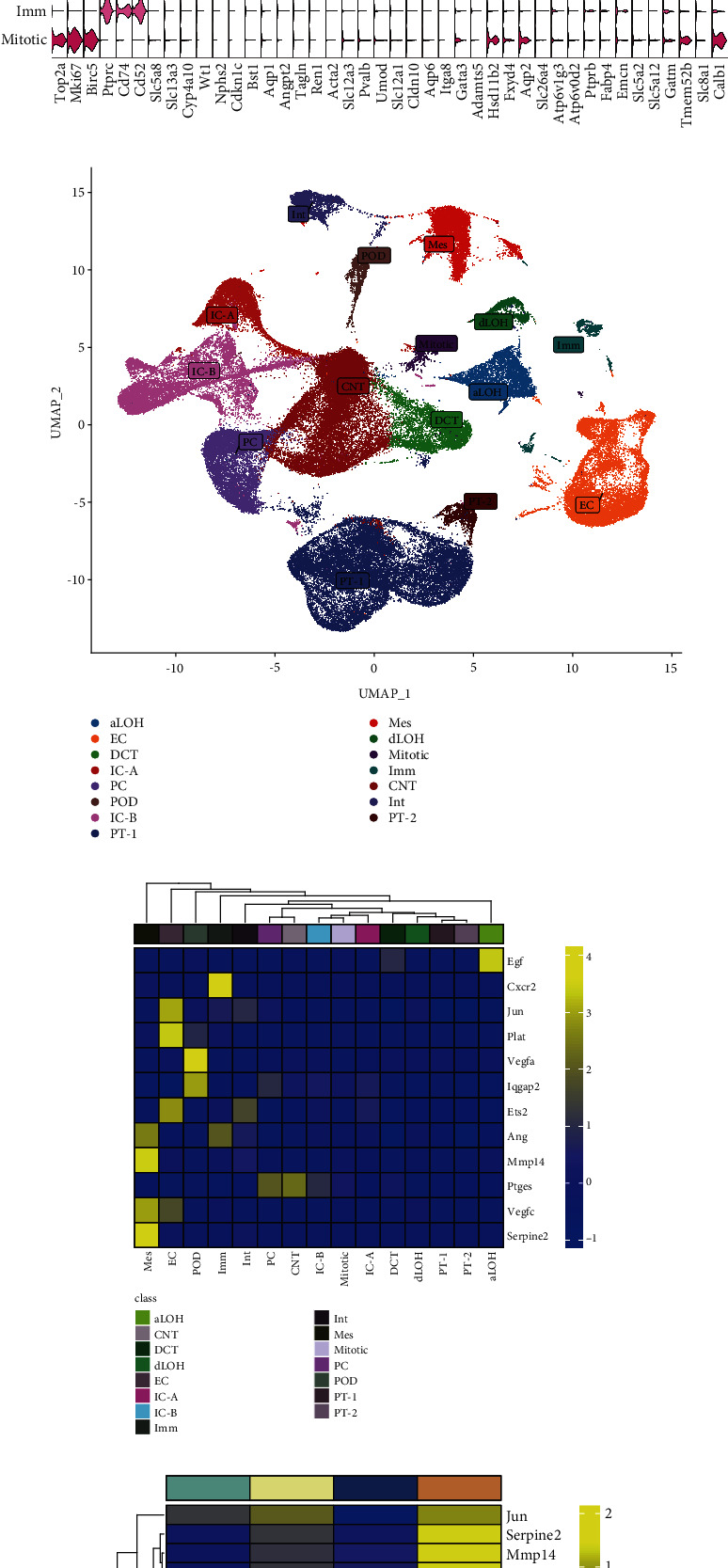
Single-cell analysis based on hub gene expression. (a) Violin plot of lineage-specific markers for each kidney cell cluster. (b) UMAP visualization of kidney cell clusters. (c) Heat map of hub gene expression in kidney cell clusters. (d) Heat map of total expression of hub genes at 06 and 12 weeks.

Several cell types were identified based on the expression of lineage-specific markers, including podocytes (POD), endothelial cells (EC), mesangial cells (Mes), interstitial cells (Int), proximal tubule (PT), descending limb of the loop of Henle (dLOH), ascending limb of the loop of Henle (aLOH), distal convoluted tubule (DCT), connecting tubule (CNT), collecting duct principal cells (PC), A-type collecting duct intercalated cells (IC-A), B-type collecting duct intercalated cells (IC-B), transition cells (Trans), immune cells (Imm), and mitotic cells (Mitotic) (Figures 5(a) and 5(b)). VEGFA and IQGAP2 exhibited high expression levels in POD, while JUN, PLAT, and ETS2 showed expression in EC. ANG, MMP14, VEGFC, and SERPINE2 were found to be expressed in Mes. Specifically, CXCR2 was uniquely expressed in Imm, PTGES was specifically expressed in CNT and PC, and EGF was observed to be specifically expressed in aLOH (Figure 5(c)). Additionally, the differential trends observed in the comparison between control and DN groups at both 06 and 12 weeks were largely consistent with the differential results obtained from our analysis based on integrated bulk RNA (Figure 5(d)).

**Figure 6:**
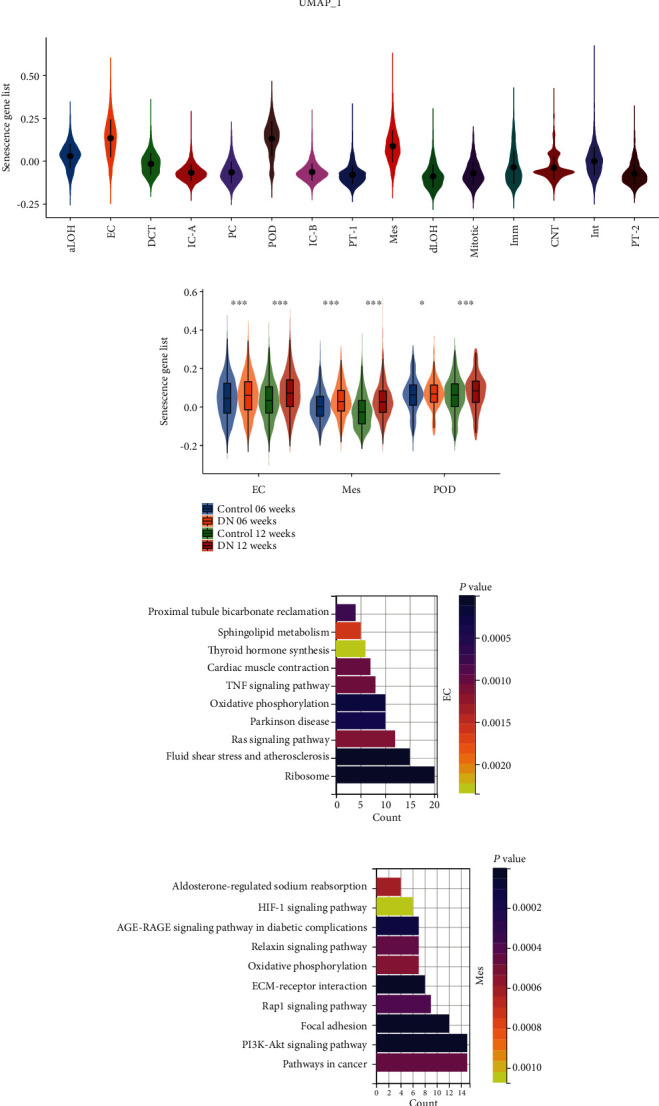
Senescence score based on hub genes. (a) UMAP visualization of senescence scores for all kidney cells. (b) Violin plot of senescence scores in kidney glomerular cell clusters (^∗^*P* value < 0.05, ^∗∗^*P* value < 0.01, and ^∗∗∗^*P* value < 0.001). (c) Bar plot of KEGG enrichment results for glomerular cell types with high and low senescence scores.

Based on the common expression patterns of these twelve genes, we generated a senescence score for all kidney cells. As depicted in Figures 6(a) and 6(b), glomerular cells (POD, EC, and Mes) exhibited a significantly higher senescence score than other kidney cell clusters. Furthermore, glomerular cells under diabetic conditions had a significantly higher senescence score than those under normal conditions, both at 06 and 12 weeks (Figure 6(c)). The KEGG analysis of DEGs between high and low senescence score groups in POD, EC, and Mes also revealed several biological changes related to senescence (Figures 6(d)–6(f)). Notably, these three cell types demonstrated a shared biological change in oxidative phosphorylation.

**Figure 7:**
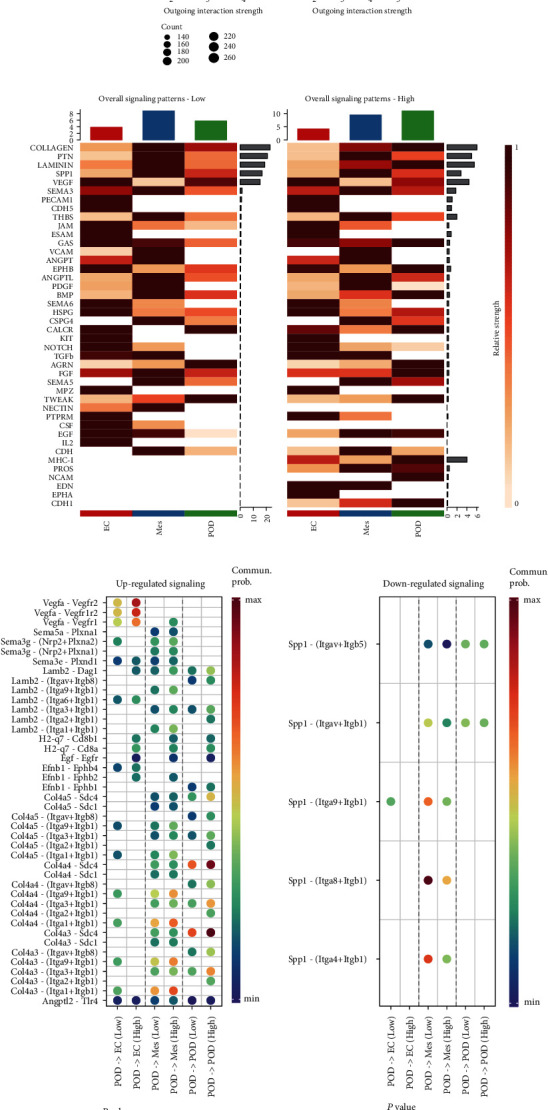
Altered cell–cell communication during senescence. (a) Change of cell–cell communication interaction strength in high and low senescence scores. (b) Change of communication pathways in high and low senescence scores. (c) Change of interactions in receptor-ligand pairs of podocytes in high and low senescence scores.

Furthermore, we employed CellChat to examine alterations in intercellular communication in glomerular cells, including secreted signaling, cell-to-cell contact, and ECM-receptor interactions. As we can see, the magnitude of change in POD was most significant in the cellular senescence environment (Figure 7(a)). In this state of senescence, the most notable altered cell communication signaling pathways include COLLAGEN, PTN, LAMININ, SPP1, and VEGF. These pathways play a role in regulating biological processes such as cell proliferation, differentiation, and migration and have been demonstrated to exert different levels of effects during the pathogenesis of chronic kidney disease (CKD) [31–35] (Figure 7(b)). The alterations in receptor-ligand pairs related to POD in the glomerulus were further identified, as shown in Figure 7(c).

### 3.5. Clinical Value Evaluation and External Database Analysis

**Figure 8:**
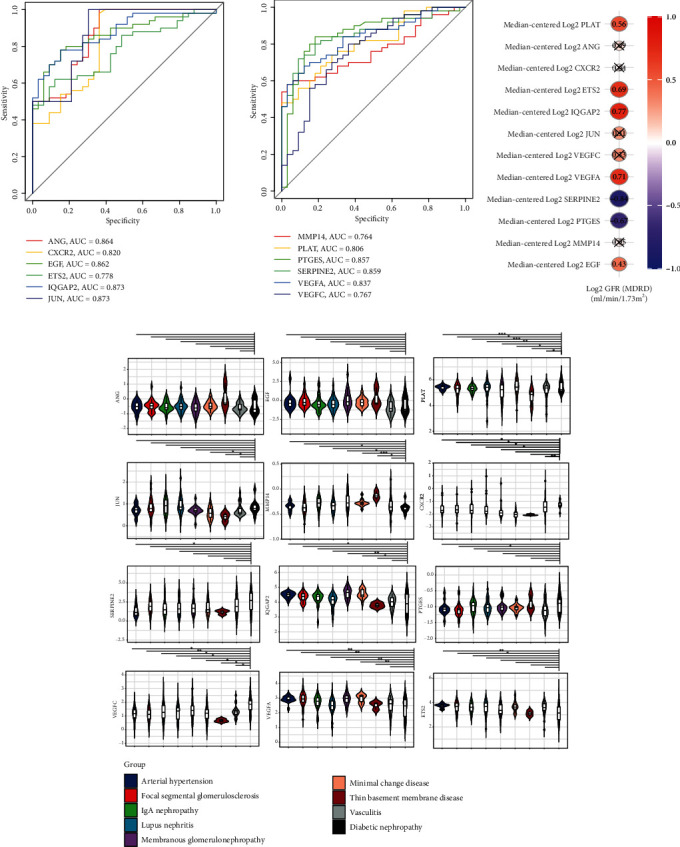
ROC analysis and external database exploration. (a) ROC analysis of hub genes in predicting diabetic glomerulopathy. (b) Correlation analysis between hub gene expression and GFR. (c) Boxplot display of expression of hub genes in various kidney diseases (^∗^*P* value < 0.05, ^∗∗^*P* value < 0.01, and ^∗∗∗^*P* value < 0.001).

ROC analysis indicated a good predictive value of the expression levels of these twelve senescence-associated genes for diabetic glomerulopathy, as shown in Figure 8(a). The areas under the curve (AUC) in the ROC analysis were 86.4% (ANG), 82.0% (CXCR2), 86.2% (EGF), 77.8% (ETS2), 87.3% (IQGAP2), 87.3% (JUN), 76.4% (MMP14), 80.6% (PLAT), 85.7% (PTGES), 85.9% (SERPINE2), 83.7% (VEGFA), and 76.7% (VEGFC). According to the analysis of the Nephroseq database, a positive correlation has been observed between the expression levels of PLAT, ETS2, IQGAP2, VEGFA, and EGF and GFR in diabetic glomerulopathy. In contrast, the expression levels of SERPINE2 and PTGES are found to be negatively associated with GFR. No significant correlation has been identified between the expression levels of ANG, CXCR2, JUN, VEGFC, and MMP14 and GFR (Figure 8(b)). Additionally, most of these genes exhibit differential expression in glomerular diseases caused by various kidney conditions (Figure 8(c)).

## 4. Discussion

Cellular senescence has been recognized as a critical determinant of aging and age-related diseases, including diabetes and its complications. Among the possible consequences of cellular senescence, DN is believed to be a condition driven largely by cellular senescence and dysfunction. The strong correlation between cellular senescence and the onset and progression of DN highlights the importance of investigating cellular senescence mechanisms in the context of DN [36–39].

In this study, we identified several potential senescence-associated biomarkers (VEGFA, IQGAP2, JUN, PLAT, ETS2, ANG, MMP14, VEGFC, SERPINE2, CXCR2, PTGES, and EGF) that may be involved in diabetic glomerulopathy from the perspective of transcriptomics. VEGFA, VEGFC, and ANG play crucial roles in regulating angiogenesis. The renal system is a complex vascular network composed of glomeruli and peritubular capillaries surrounding the tubules, which is fundamental for normal renal cell function. The maintenance of renal vascular integrity relies on a delicate balance between proangiogenic factors and antiangiogenic factors, and disruption of this balance is implicated in various kidney diseases, including DN [40]. EGF, JUN, and ETS2 are involved in cell proliferation and growth. EGF is a growth factor involved in various biological processes. It has been shown that EGF protects the kidney by promoting cell proliferation and reducing apoptosis and fibrosis [41]. Studies have shown that inhibition of the JUN expression or activity can attenuate the progression of DN, ameliorate pathological damage to the kidneys, and enhance renal function [42]. According to current knowledge, ETS2, a gene encoding a transcription factor located on human chromosome 2, has not been extensively studied in DN. The ETS2 protein plays an important role in a variety of biological processes, including embryonic development, cell proliferation and differentiation, apoptosis, angiogenesis, and immune response. ETS2 deficiency may lead to endothelial dysfunction and injury, which accelerates the progression of atherosclerosis and promotes cardiovascular disease [43]. CXCR2 and PTGES are closely tied to inflammation and immune responses. It has been shown that in DN, the expression level of CXCR2 increases, and it participates in the occurrence and progression of the disease by regulating inflammatory and fibrotic processes [44]. PTGES contributes to the pathological processes of DN through various mechanisms, such as increasing GFR, promoting the proliferation of renal tubule cells, and inducing inflammatory responses [45]. IQGAP2 and MMP14 have a connection to cell adhesion and migration. IQGAP2 is expressed in multiple organs and has been linked to the development of metabolic diseases and tumors [46–48]. However, its role in DN and the specific mechanisms need to be investigated further. In DN, MMP14 expression levels are elevated, and it participates in pathological processes such as glomerulosclerosis and accumulation of the extracellular matrix [49, 50]. In patients with DN, platelet aggregation, elevated plasma fibrinogen levels, and vascular endothelial cell injury are factors that lead to abnormal activation of the coagulation and fibrinolysis systems [51]. PLAT and SERPINE2 are mainly coagulation- and fibrinolysis-related genes. The abnormal activation of the coagulation and fibrinolysis systems can lead to thrombosis and microcirculation disorders, which can affect renal function [51]. ROC analysis of the expression levels of these genes also suggests that they may be of good clinical value for DN.

Furthermore, we have delineated discrete molecular subtypes of senescence within DN by analyzing the shared expression patterns of the aforementioned genes. Aging leads to immune system decline, known as immunosenescence [52]. With increasing age, the numbers and functions of various immune cells including T cells, B cells, natural killer cells, and monocytes in the human body decrease to varying degrees. Moreover, the cytokines and chemokines in the elderly that regulate immune cell activity also undergo changes, further affecting the functionality of the immune system. There are biological differences in the cellular immune response, inflammatory response, cell cycle, and metabolic regulation between the two senescence molecular subtypes (C1 and C2) that we have identified in our study. At the same time, subtype C2 is associated with decreased activation of various immune cells, such as activated NK cells, monocytes, neutrophils, and activated mast cells. The above findings may provide new ideas for revealing the senescence heterogeneity of DN, as well as individualized treatment.

We also investigated the cellular cluster localization of these genes in the kidney from a single-cell perspective and validated their expression in DN. The senescence microenvironment was also further evaluated. In DN, glomerular damage and aging are more obvious [53]. The KEGG analysis indicated that oxidative phosphorylation was involved in both high and low senescence score glomerular cell clusters. Oxidative phosphorylation is a process by which ATP is produced within cells and has been associated with aging and DN [54]. Research has shown that oxidative phosphorylation is related to mitochondrial dysfunction and apoptosis during the aging process, making it an important mechanism of aging [55]. In DN, hyperglycemia leads to an increase in mitochondrial oxidative phosphorylation and leads to mitochondrial dysfunction and excessive production of free radicals, thereby inducing oxidative damage and inflammation in kidney tissue [56].

DN is a chronic complication of diabetes that is characterized by increased levels of albuminuria [57]. This occurs due to damage to the glomerular filtration barrier caused by diabetes. POD, which are essential components of this barrier, play a critical role in maintaining its structural and functional integrity. The loss or injury of POD is strongly associated with the progression of DN and the severity of albuminuria [58]. In diabetes, POD can become dysfunctional or injured due to various pathogenic cues such as hyperglycemia, oxidative stress, vasoactive mediators, cytokines, and growth factors, which activate the signaling of cellular senescence [4]. Our results also indicated that POD exhibit the most significant magnitude of changes under senescence, as assessed jointly by the hub genes we identified. In addition, we further identified potential communication pathways and ligand-receptor interactions that have been largely mentioned previously in relation to the pathogenesis of DN. These demonstrated the true reliability of these senescence-associated genes to reflect DN.

Our study has several limitations. Firstly, the lack of clinical information for each kidney biopsy sample limits our ability to evaluate the degree of association between gene expression levels and clinical features. Additionally, further research is needed to explore the methods for associating subgroups with important clinical and laboratory indicators. Secondly, additional molecular biology experiments are necessary to investigate the specific mechanisms of senescence-associated genes in DN. Thirdly, we did not investigate further the mechanism of these genes in other kidney diseases.

## 5. Conclusion

In this study, we ultimately identified twelve representative senescence-associated genes and molecular subtypes that might provide new insights into the assessment, management, and personalized treatment of DN.

## Data Availability

This study analyzed data obtained from the publicly accessible databases. Data was collected from the GEO and Nephroseq databases.

## Supplementary Materials

Supplementary Materials Attachment 1: dot plot of GO enrichment results (BP, MF, and CC) for DEGs.
