# Supplementary figures and images for: Identification of Senescence-Associated Biomarkers in Diabetic Glomerulopathy Using Integrated Bioinformatics Analysis
Source: J Diabetes Res. 2024 Jan 23;2024:5560922. doi: 10.1155/2024/5560922 (PMC10827377; doi:10.1155/2024/5560922)

Tag

A

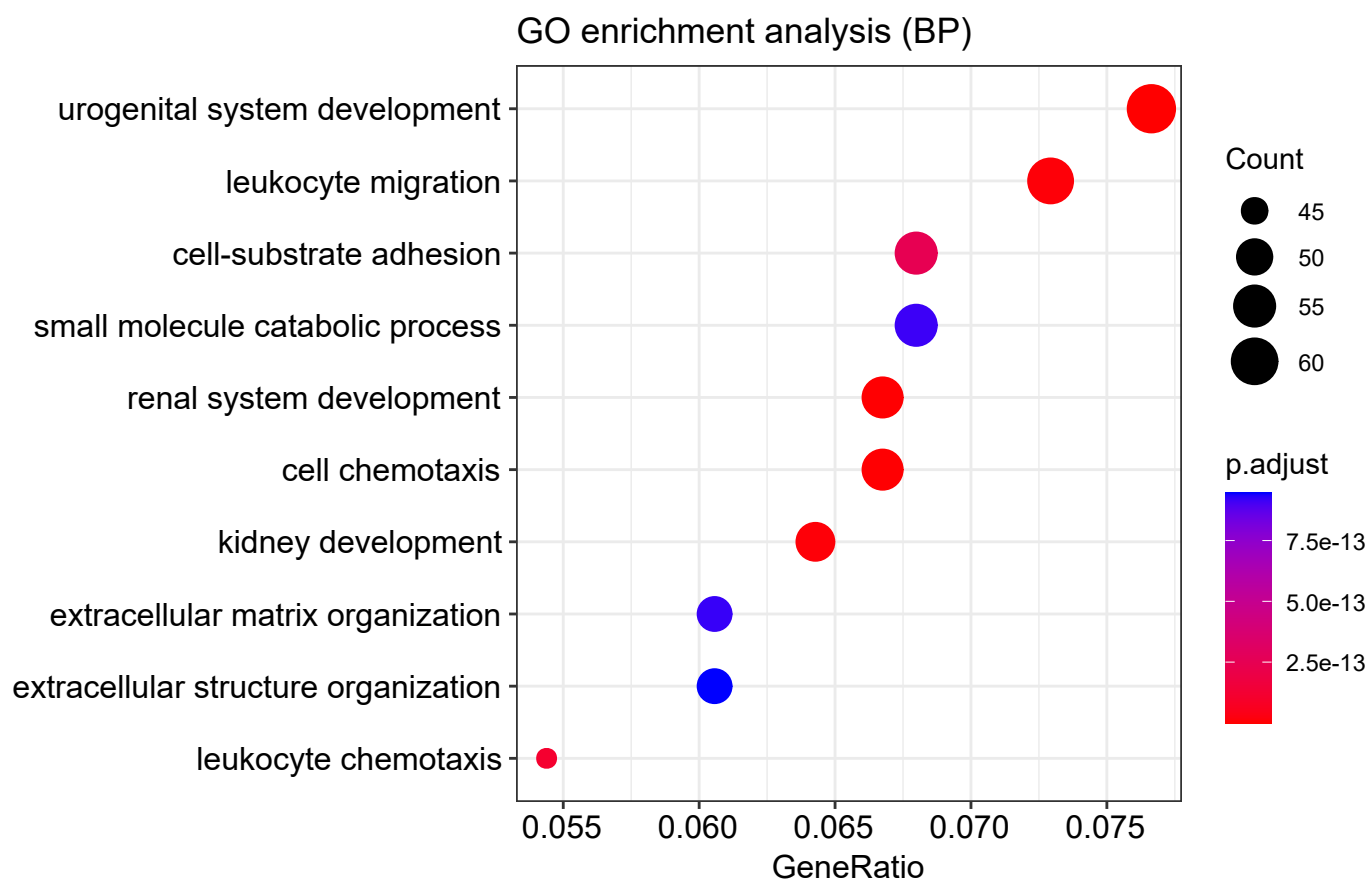

B

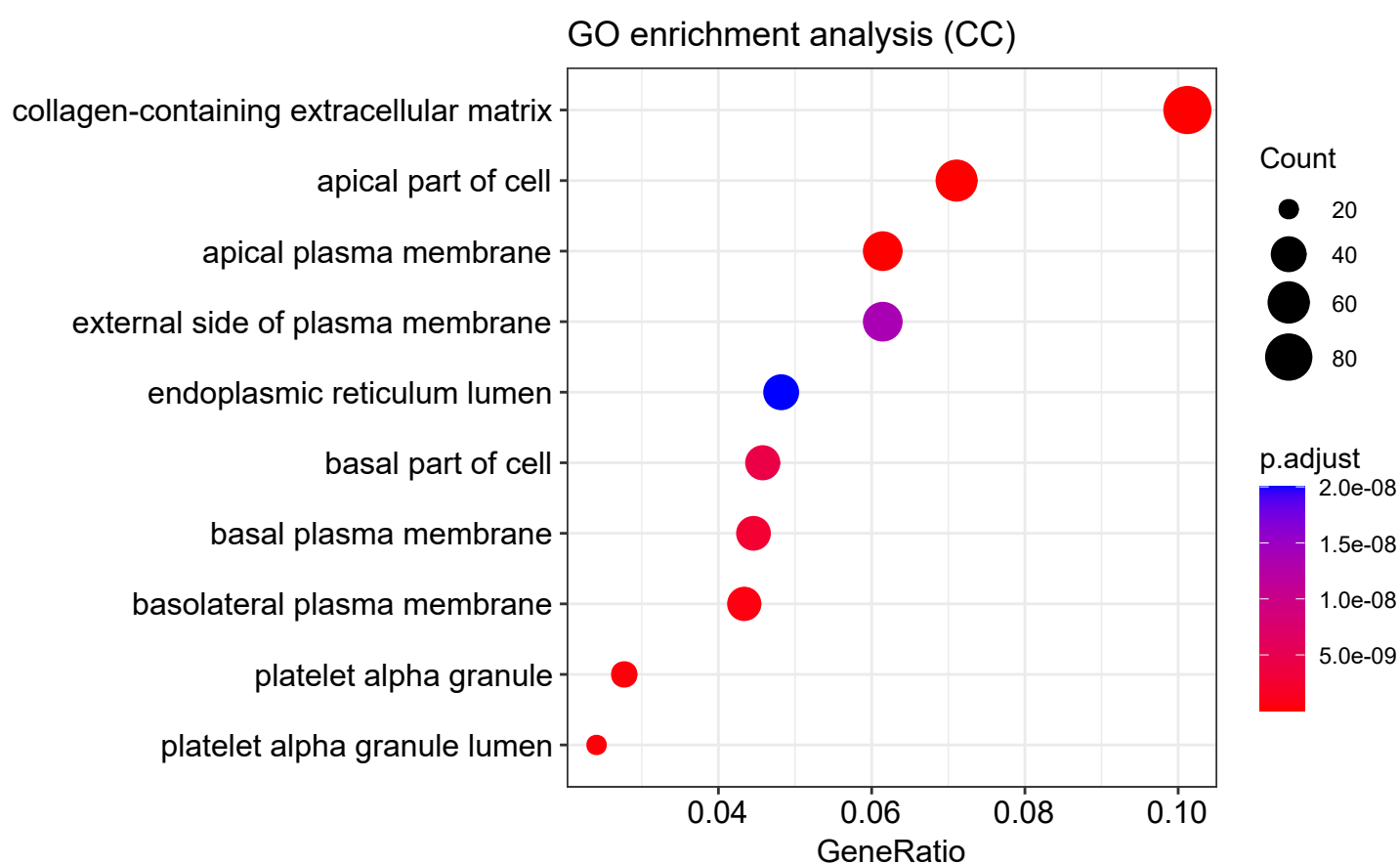

C

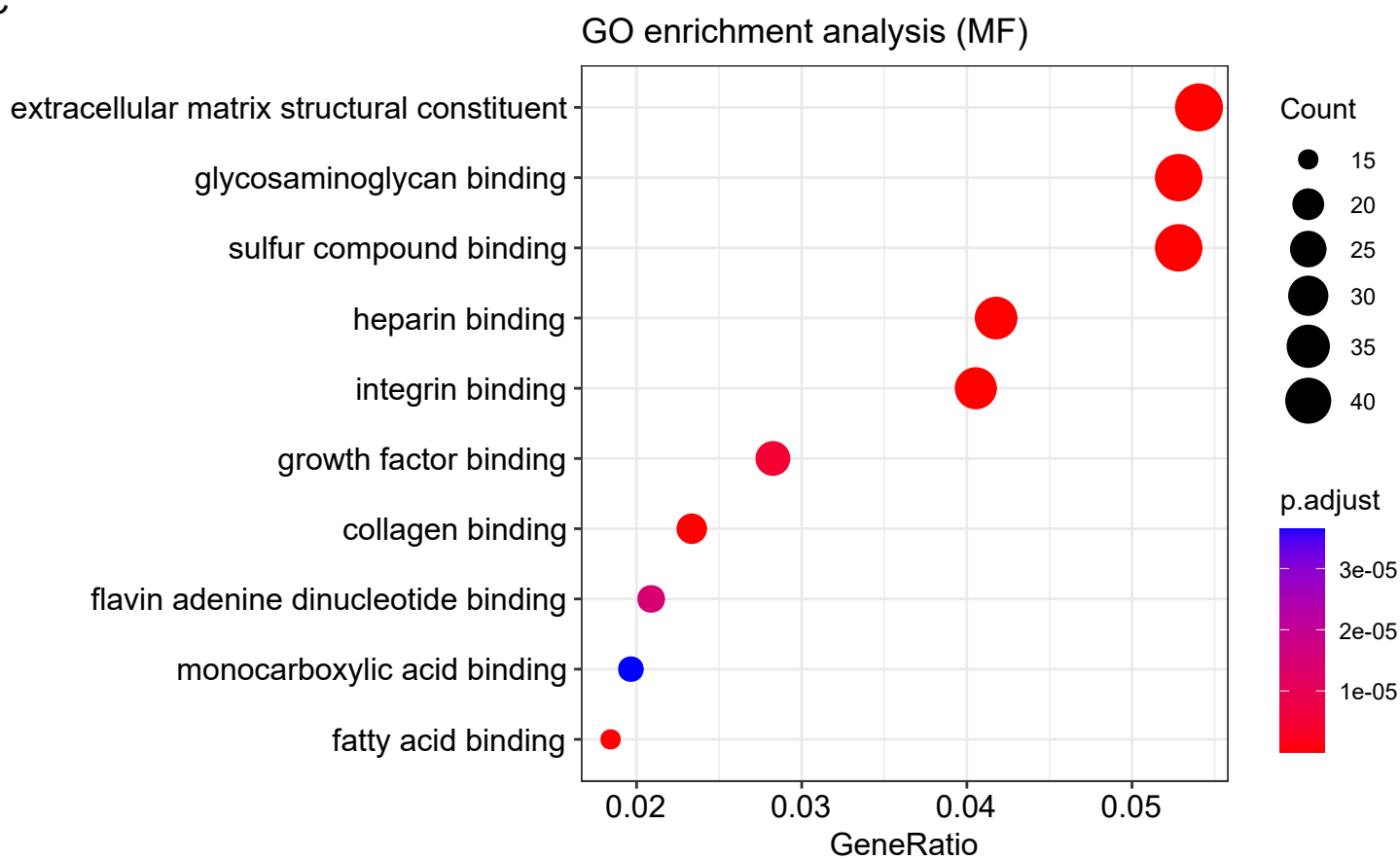

Supplement: Supplementary Materials — Attachment 1: dot plot of GO enrichment results (BP, MF, and CC) for DEGs. [file 5560922.f1.pdf]
